# A simple and rapid method for measuring *α*-D-phosphohexomutases activity by using anion-exchange chromatography coupled with an electrochemical detector

**DOI:** 10.7717/peerj.1517

**Published:** 2016-01-05

**Authors:** Xiaochen Jia, Jian Kang, Heng Yin

**Affiliations:** 1 Dalian Institute of Chemical Physics, Chinese Academy of Sciences, Dalian, China; 2 University of Chinese Academy of Sciences, Beijing, China; 3 Department of Biochemistry and Molecular Biology, Dalian Medical University, Dalian, China

**Keywords:** HPAEC-PAD, N-acetylglucosamine-phosphate mutase, N-acetylglucosamine-1-phosphate, N-acetylglucosamine-6-phosphate, α-D-phosphohexomutases

## Abstract

The interconversion of hexose-6-phosphate and hexose-1-phosphate can be directly analyzed by high-performance anion-exchange chromatography coupled with an electrochemical detector (HPAEC-PAD). Thus, this method can be used to measure the activities of N-acetylglucosamine-phosphate mutase (AGM), glucosamine-phosphate mutase (GlmM) and phosphoglucomutase (PGM), which are the members of *α*-D-phosphohexomutases superfamily. The detection limits were extremely low as 2.747 pmol, 1.365 pmol, 0.512 pmol, 0.415 pmol, 1.486 pmol and 0.868 pmol for N-acetylglucosamine-1-phosphate (GlcNAc-1-P), N-acetylglucosamine-6-phosphate (GlcNAc-6-P), glucosamine-1-phosphate (GlcN-1-P), glucosamine-6-phosphate (GlcN-6-P), glucose-1-phosphate (Glc-1-P) and glucose-6-phosphate (Glc-6-P), respectively. By employing HPAEC-PAD, activities of *At*AGM (AGM from *Arabidopsis thaliana*) on these six phosphohexoses can be detected. The *K_m_* of *At*AGM on Glc-1-P determined by HPAEC-PAD was 679.18 ± 156.40 µM, which is comparable with the *K_m_* of 707.09 ± 170.36 µM detected by traditional coupled assay. Moreover, the activity of *Mt*GlmM (GlmM from *Mycobacterium tuberculosis*) on GlcN-6-P tested by HPAEC-PAD was 7493.40 ± 309.12 nmol∕min ⋅ mg, which is much higher than 288.97 ± 35.28 nmol∕min ⋅ mg obtained by the traditional coupled assay. Accordingly, HPAEC-PAD is a more rapid and simple method than the traditional coupled assays given its high specificity and sensitivity, and will certainly bring convenience to further research of *α*-D-phosphohexomutases.

## Introduction

N-acetylglucosamine (GlcNAc) is an essential sugar involved in various cellular processes. Generally, uridine di-phosphate (UDP)-GlcNAc is the active form of GlcNAc *in vivo*, and synthesized in a four-step cytoplasmic biosynthesis pathway, which is known as hexosamine pathway (Milewski, Gabriel & Olchowy, 2006; Piacente et al., 2014). The hexosamine pathway is a branch of glycolysis. It is associated with posttranslational protein modification by glycosylation and involved in the synthesis of glycolipids, proteoglycans, and glycosylphosphatidylinositol anchors (Vosseller et al., 2002; Wells, Vosseller & Hart, 2001; Zachara & Hart, 2004). The hexosamine pathway is a survival feature of neoplastic cells, plays a prominent role during tumourigenesis, and can be exploited therapeutically to target cancer cells (Vasseur & Manie, 2015). The hexosamine pathway is also essential for the growth of pathogenic bacteria, and can be used as a potential excellent target for anti-bacteria drugs (Li et al., 2012).

**Figure 1:**
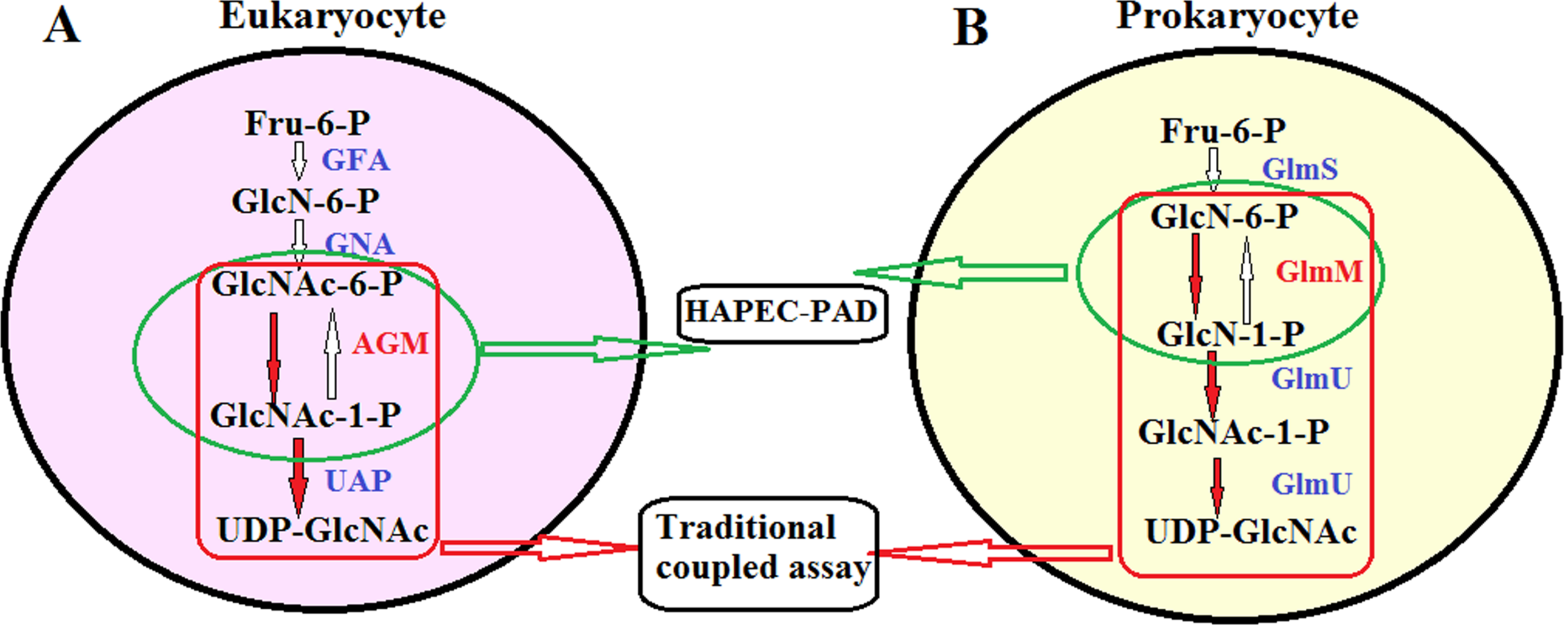
The hexosamine pathway in eukaryocyte and prokaryocyte. (A) The pathway in eukaryocyte was catalyzed by GlcN-6-P synthase (GFA), GlcN-6-P acetyltransferase (GNA), N-acetylglucosamine-phosphate mutase (AGM) and UDP-GlcNAc pyrophosphorylase (UAP) respectively. (B) The pathway in prokaryocyte was catalyzed by glutamine fructose-6-phosphate transferase (GlmS), phosphoglucosamine mutase (GlmM), glucosamine-1-phosphate acetyltransferase/N-acetylglucosamine-1-phosphate uridyltransferase (GlmU) respectively. The activity of AGM and GlmM could be detected by HAPEC-PAD method or traditional coupled assay.

In living organisms, there are three variations of hexosamine pathway according to the different reaction sequence and enzymes, which are eukaryotic (Fig. 1A), prokaryotes (Fig. 1B) and mimivirus pathways (Piacente et al., 2014). The hexosamine pathway in mimivirus follows the eukaryotic-like strategy, but also shares some properties with prokaryotic pathway (Piacente et al., 2014). The enzymes in hexosamine pathway have been well studied except N-acetylglucosamine-phosphate mutase (AGM) and glucosamine-phosphate mutase (GlmM) (Fang et al., 2013; Milewski, Gabriel & Olchowy, 2006). The main reason is the limitation of methodology on *α*-D-phosphohexomutases activity.

AGM, GlmM and phosphoglucomutase (PGM) all belong to the *α*-D-phosphohexo-mutases superfamily (Shackelford, Regni & Beamer, 2004). Despite the differences in substrate specificity, the catalytic properties in this superfamily are nearly the same. They catalyze the reversible conversion of hexose-6-P to hexose-1-P via a bisphosphorylated sugar intermediate. Active enzyme is phosphorylated at a conserved serine residue and binds one Mg^2+^ ion (Shackelford, Regni & Beamer, 2004). Reversible conversion of hexose-1-P to hexose-6-P is difficult to be detected by a conventional method.

Traditionally, the activity of *α*-D-phosphohexomutase was determined by a coupled enzyme system. PGM catalyze the interconvertion of glucose-1-phosphate (Glc-1-P) and Glc-6-P. The normal way to detect the activity of PGM was using Glc-1-P as substrates, glucose-6-phosphate dehydrogenase (G6PDH) as coupled enzyme, by detect the increasing absorbance values of NADH at 340 nm (Nishinari et al., 2012; Wang & Zhang, 2010). This method was convenience and simple because G6PDH was commercially available and cost-effective. However, the detection of the reverse reaction which uses Glc-6-P as a substrate is still impossible.

GlcNAc-6-P was always used as a substrate for detecting the AGM activity, and GlcNAc-1-P uridylyltransferase (UAP), the fourth enzyme in hexosamine pathway, was used as a coupled enzyme (Boles et al., 1994; Greig et al., 2007; Jolly et al., 2000) (Fig. 1A). The detection of GlmM activity was carried out by using GlcN-6-P as substrate, and coupled with the third and forth enzyme GlmU (Li et al., 2012; Li et al., 2011) (Fig. 1B). However, this coupled method was very complex and tedious, and especially UAP and GlmU were commercial unavailable. Furthermore, the most noticeable limitation of the traditional coupled assay is impossible to detect the reverse reaction, which using GlcNAc-1-P or GlcN-1-P as substrate.

In order to overcome the limitations of the traditional coupled assay on the determination of the activity of *α*-D-phosphohexomutases, a simple and convenient method which can detect the interconversion of hexose-6-P and hexose-1-P directly is urgently needed.

High-performance anion-exchange chromatography (HPAEC) has been used for the quantitation of carbohydrates (Kazlowski, Pan & Ko, 2015; Yang, Hsieh & Lin, 2015) because its strong anion-exchange property allows the highly selective separation of carbohydrates. It has been tried to detect and separate the phosphohexoses directly by HPAEC for decades. In previous studies, Jolly et al. (1999) detected glucosamine phosphates by HPAEC with a post-column derivatization. detected glucose phosphates by HPAEC with ^14^C labeled method. Naught & Tipton, (2005). However, they all needed radiochemical detector or fluorescence detector, and the phosphohexoses pretreatment were complex and expensive. Recently, several phosphohexoses had been successfully separated by HPAEC (Jeong et al., 2007; Marcellin et al., 2009), but the separation of GlcNAc-1-P and GlcNAc-6-P is still unsuccessful. And also, there is no simple and convenient way to determine the activity of *α*-D-phosphohexomutases directly.

In this study, we developed a method by using HPAEC-PAD to separate phosphohexoses, which can be helpful to determine *α*-D-phosphohexomutases activity, especially AGM. Considering that AGM also has the ability to convert other phosphohexose (Boles et al., 1994; Fang et al., 2013; Greig et al., 2007; Hofmann, Boles & Zimmermann, 1994), the interconvertion of GlcNAc-6-P and GlcNAc-1-P, Glc-6-P and Glc-1-P, as well as GlcN-6-P and GlcN-1-P was investigated. In our knowledge, this is the first report to detect the AGM activity directly. This method also can be used to determine the activities of GlmM and PGM, which convert the interconvertion of GlcN-6-P and GlcN-1-P, Glc-6-P and Glc-1-P.

## Materials and Methods

### Materials

Glc-1-P, Glc-6-P, glucose-1,6-bisphosphate (Glc-1,6-2P) and GlcN-6-P were purchased from Sigma-Aldrich. GlcNAc-6-P, GlcNAc-1-P and GlcN-1-P were purchased from Santa Cruz Biotechnology. Double distilled water was used for HPLC, and all other solvents and chemicals were HPLC grade.

### Chromatography

The HPAEC system consists of a Dionex Bio-LC gradient pump with GM-3 (4 mm) gradient mixer, CarboPac PA-100 column (4 × 250 mm), and an electrochemical detector with AgCl as reference electrode. The waveform was carbohydrates (standard Quad), the following pulse potentials were used for detection: *t* = 0 s, *E* = 0.10 v; *t* = 0.20 s, *E* = 0.10 v; *t* = 0.40 s, *E* = 0.10 v; *t* = 0.41 s, *E* = − 2.00 v; *t* = 0.42 s, *E* = − 2.00 v; *t* = 0.43 s, *E* = 0.60 v; *t* = 0.44 s, *E* = − 0.10 v; *t* = 0.50 s, *E* = − 0.10 v. The sample injection volume is 20 µL and column oven temperature is maintained at 30 °C.

### Elute conditions

The phosphohexoses should be eluted after 10 min because there were several interference peaks in the enzyme reaction mixture before 10 min on chromatograms. Thus, high pH elution was carried out in 100 mM sodium hydroxide under gradient conditions using a 80–720 mM sodium acetate gradient over 20 min at a flow rate of 0.5 ml/min.

The details of gradient conditions were 0–5 min, 80 mM sodium acetate; 5–15 min, 80–720 mM sodium acetate; 15–18 min, 720 mM sodium acetate; 18–20 min, 80 mM sodium acetate in 100 mM sodium hydroxide, 0.5 ml/min.

### Expression and purification of *At*AGM and *Mt*GlmM

*E. coli* cells (*At*AGM::Amp) were grown exponentially at 37 °C in LB medium with Amp. When the optical density (OD) of the culture reached 0.8, IPTG was added at a final concentration of 1 mM, and growth was continued for 12 h at 16 °C. Harvested cells were disrupted by sonication, centrifugated at 20,000 g for 30 min; the resulting supernatant was loaded onto Ni-NTA column (Qiagen, Hilden, Germany) for purification.

*Mt*GlmM and *Mt*GlmU were expressed and purified according to our previous studies (Li et al., 2012; Li et al., 2011).

### *At*AGM assay

The activities of *At*AGM (N-acetylglucosamine-phosphate mutase, from *Arabidopsis thaliana*) on different phosphohexoses, were tested in the same reaction mixture as described below. 154.80 pmol (10 µg) *At*AGM or boiled enzymes, were incubated in a substrate buffer (300 µl) consisting of 20 mM PBS, pH 7.0, 10 mM MgSO_4_, and 20 µM Glc-1,6-2P and 0.1 mM different phosphohexose (GlcNAc-1-P, GlcNAc-6-P, GlcN-1-P, GlcN-6-P, Glc-1-P, Glc-6-P) (Fang et al., 2013; Hofmann, Boles & Zimmermann, 1994) for 30 min at 30 °C. The reaction was terminated in boiling water and 200 µl of 0.2 M NaOH were added for HPAEC-PAD analysis.

The Steady-state kinetics of *At*AGM was analyzed by two methods using Glc-1-P as substrates. The HPAEC-PAD method was carried out by using 56.66 pmol *At*AGM or boiled enzymes, incubated for 10 min at 30 °C in a substrate buffer as described above, with varying concentrations of Glc-1-P (60–8,000 µM). Then, the assay was terminated immediately in boiling water and 200 µl of 0.2 M NaOH were added for HPAEC-PAD analysis.

The traditional coupled assay was carried out in a 200 µl reaction volume containing 20 mM PBS, pH 7.0, 10 mM MgSO_4_, 20 µM Glc-1,6-2P, a range of concentrations of Glc-1P (60–7,000 µM), 1 mM NAD^+^ and 2 units of G6PDH (Anasontzis et al., 2014; Fang et al., 2013; Kim et al., 2014). The reaction was started by the addition of 56.66 pmol *At*AGM and incubated for 10 min at 30 °C. The amount of NADH produced was measured using a micro plate reader at 340 nm (BioTek, Gene 5).

All the experiments had repeated three times, and the *K_m_* and *V*_max_ values were calculated by Origin 7.5.

### *Mt*GlmM assay

*Mt*GlmM (glucosamine-phosphate mutase, from *Mycobacterium tuberculosis*) activity was determined by two methods.

The traditional coupled assay was conducted in a coupled enzyme system with *Mt*GlmU, the third- and forth-step enzymes in hexosamine pathway in *Mycobacterium tuberculosis* (Li et al., 2011) (Fig. 1B). The GlcN-1-P converted from GlcN-6-P by the mutase was quantitatively converted into UDP-GlcNAc in the presence of purified *Mt*GlmU in this coupled assay (Li et al., 2012) (Fig. 1B). The 50 µl reaction mixture contained 50 mM Tris–HCl, pH 8.0, 2.5 mM MgCl_2_, 1 mM GlcN-6-P, 0.6 mM acetyl-CoA, 0.2 mM Glc-1,6-2P, purified *Mt*GlmU (3.5 µg) and purified *Mt*GlmM (0.18 µg). The reaction was incubated at 37 °C for 20 min and terminated by adding 50 µl of stop solution containing 50 mM Tris–HCl, pH 7.5, 6 M guanidine hydrochloride. The mixture was then incubated for 10 min by the addition of 50 µl of Ellman’s reagent solution containing 0.2 mM DTNB in the buffer with 50 mM Tris–HCl, pH 7.5, and 1 mM EDTA.TNB, the product generated from the reaction of CoA-SH and DTNB, was monitored at 405 nm by Benchmark Plus plate reader (Thermo Fisher Scientific, Waltham, MA, USA).

*Mt*GlmM activity was analyzed using HPAEC-PAD by detecting the interconversion of GlcN-6-P and GlcN-1-P directly. The purified *Mt*GlmM (0.18 µg) was incubated at 37 °C for 20 min in a substrate buffer (50 µl) consisting of 1 mM GlcN-6-P (or GlcN-1-P) as substrate with 50 mM Tris–HCl, pH 8.0, 2.5 mM MgSO_4_, and 0.2 mM Glc-1,6-2P. The reaction was terminated immediately in boiling water and 500 µl of 0.2 M NaOH were added for HPAEC-PAD analysis.

## Results and Discussion

### The separation of standards

**Figure 2:**
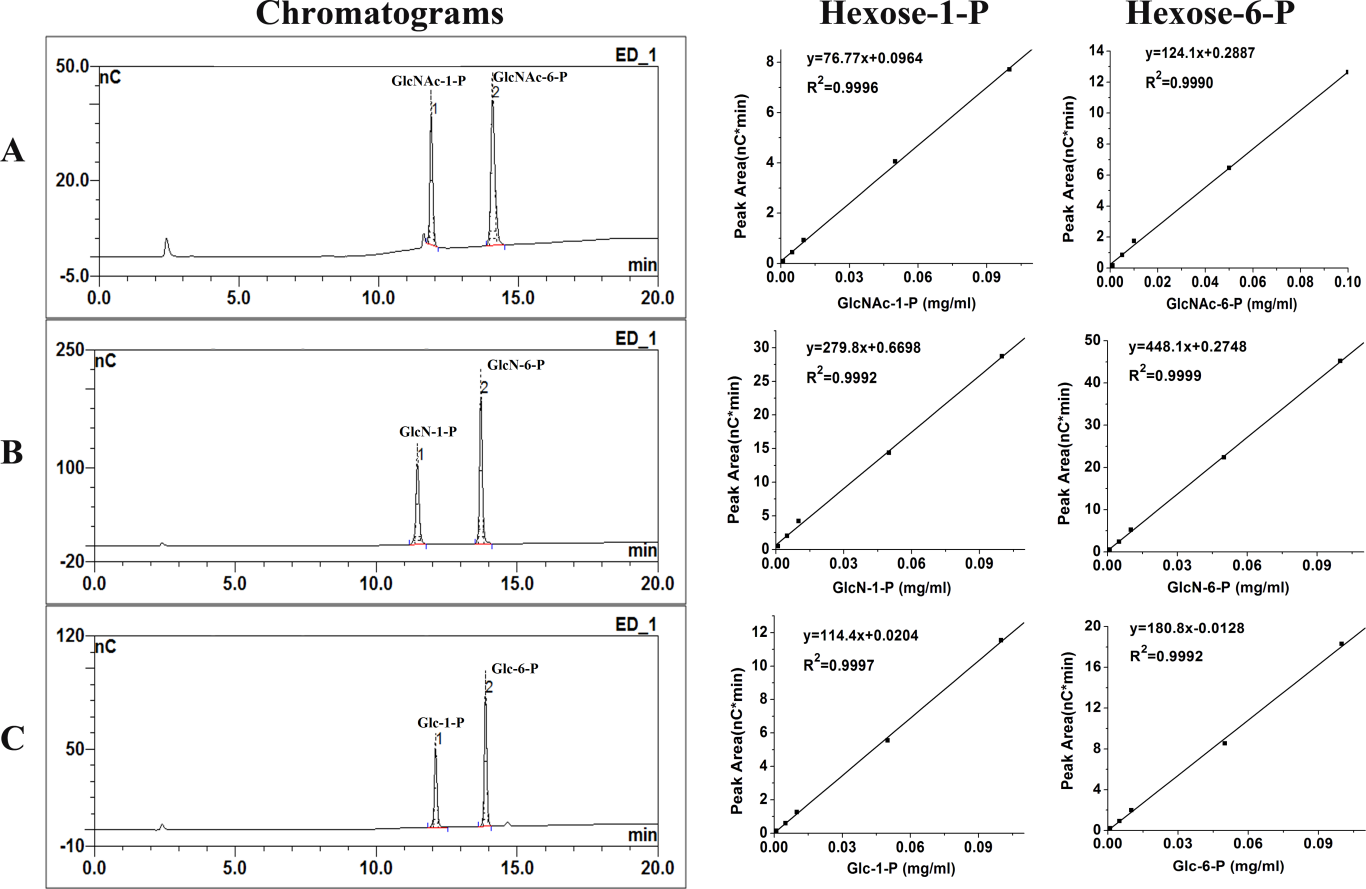
Chromatograms of calibration standards and their standard curves. Under the 80–720 mM sodium acetate gradient over 20 min at a flow rate of 0.5 ml/min, the calibration standards of 0.05 mg/ml GlcNAc-1-P, GlcNAc-6-P (A) ; 0.05 mg/ml GlcN-1-P, GlcN-6-P (B); 0.05 mg/ml Glc-1-P, Glc-6-P (C) were separated excellent on HPAEC-PAD chromatograms. Their standard curves were shown behind their chromatograms respectively; “nC” means PAD response.

Under the elute conditions described in ‘Materials and Methods’, the retention times of GlcNAc-1-P, GlcNAc-6-P, GlcN-1-P, GlcN-6-P, Glc-1-P and Glc-6-P were 12.17 min, 14.30 min, 11.55 min, 13.75 min, 12.05 min and 13.72 min, respectively (Figs. 2A–2C). Hexose-1-P and hexose-6-P were separated excellent by HPAEC-PAD.

The detection limits are different for different phosphohexoses. The detection limits were 2.747 pmol, 1.365 pmol, 0.512 pmol, 0.415 pmol, 1.486 pmol and 0.868 pmol for GlcNAc-1-P, GlcNAc-6-P, GlcN-1-P, GlcN-6-P, Glc-1-P and Glc-6-P, respectively. The linearity of all the phosphohexoses was excellent (2–400 µM, *R*^2^ > 0.999) as shown in Fig. 2. And HPAEC-PAD response was linearity in the range of concentration 1–15,000 µM (*R*^2^ > 0.99).

Therefore, HPAEC-PAD method can be used to identify phosphohexose and determine the activity of *α*-D-phosphohexomutases by detecting the substrate consuming and product increasing at the same time.

### AGM assay

**Figure 3:**
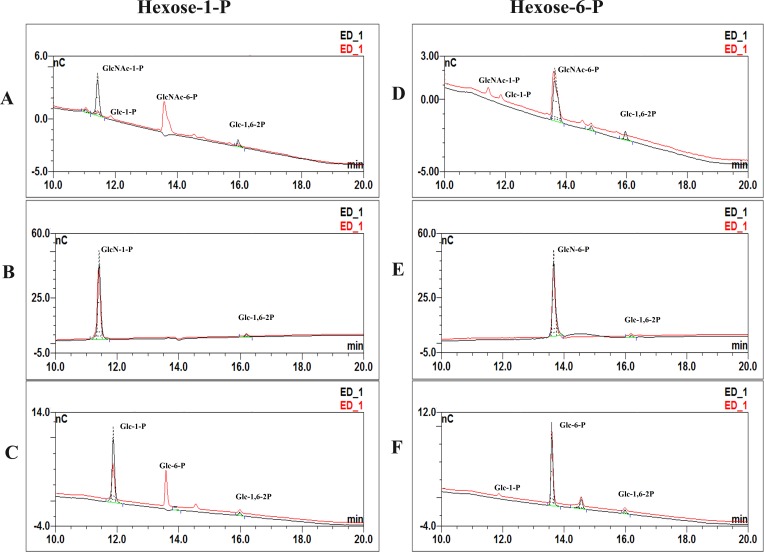
*At*AGM activity on different substrates was assayed by HPAEC-PAD method. 154.80 pmol *At*AGM or boiled enzymes, were incubated for 30 min at 30 °C in a substrate buffer (300 µl) consisting of 20 mM PBS, pH 7.0, 10 mM MgSO4, and 20 µM glucose-1,6-bisphosphate (Glc-1,6-2P). And using GlcNAc-1-P (A), GlcN-1-P (B), Glc-1-P (C), GlcNAc-6-P (D), GlcN-6-P (E), and Glc-6-P (F) as substrate respectively (the final concentration is 0.1 mM). The black or red lines means the mixture after reaction, incubated with the boiled enzyme or *At*AGM , respectively; “nC” means PAD response.

The chromatograms of the reaction mixtures of *At*AGM incubated with different substrates (GlcNAc-1-P, GlcNAc-6-P, GlcN-1-P, GlcN-6-P, Glc-1-P and Glc-6-P) were shown in Fig. 3, including the chromatograms before (incubated with boiled enzyme) and after the reaction. The consuming of substrates and the producing of the products could be observed well in the chromatograms. The results showed that *At*AGM indeed had the ability to convert these six phosphohexoses.

**Figure 4:**
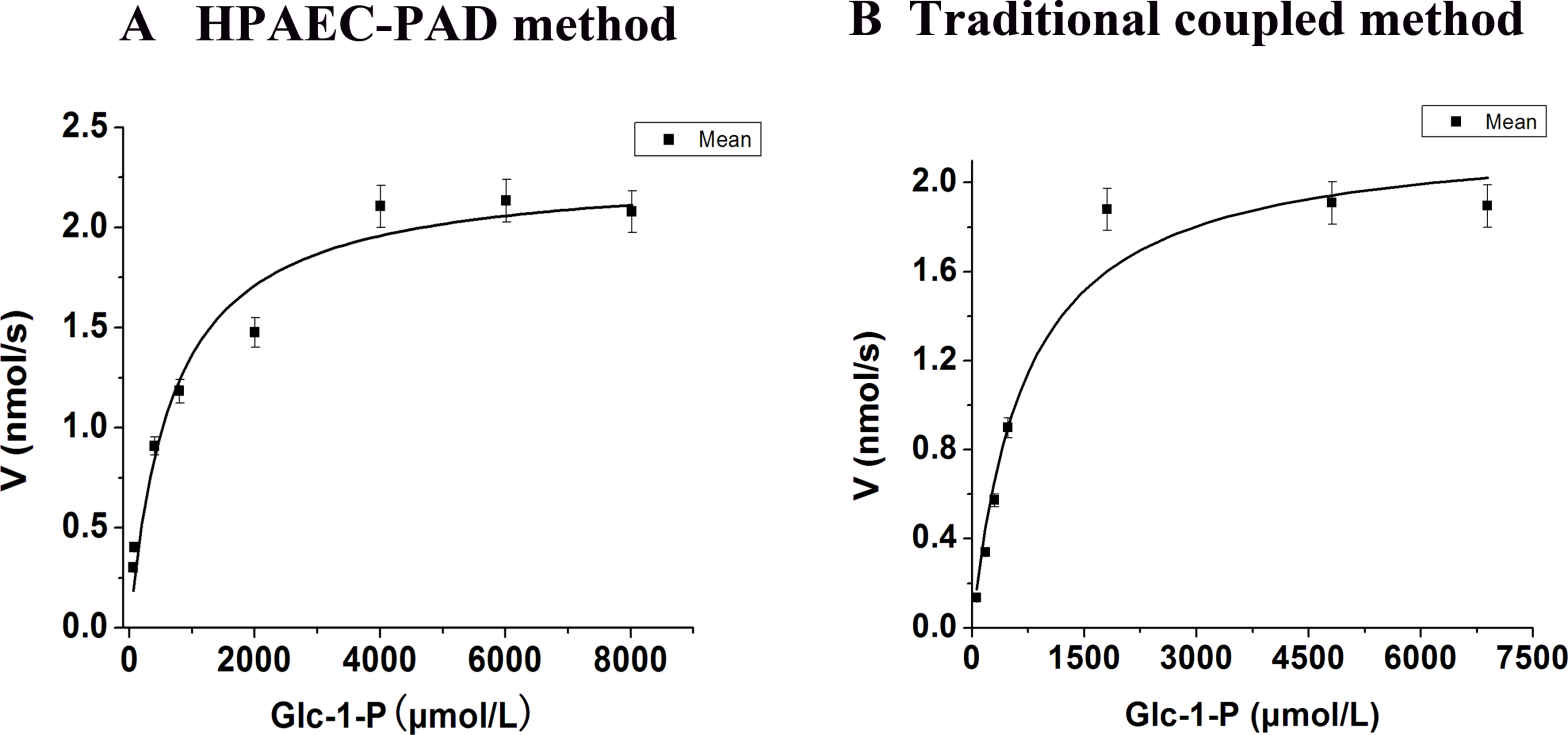
Steady-state kinetics of *At*AGM was analyzed by using Glc-1-P as substrates by two methods. (A) The HPAEC-PAD method was carried out by using 56.66 pmol *At*AGM or boiled enzymes, incubated for 10 min at 30 °C in a substrate buffer (300 µl) consisting of 20 mM PBS, pH 7.0, 10 mM MgSO4, and 20 µM glucose-1,6-bisphosphate (Glc-1,6-2P), with varying concentrations of Glc-1-P (60–8,000 µM). (B)The traditional coupled assay was carried out in a 200 µl reaction volume containing 20 mM PBS, pH 7.0, 10 mM MgSO_4_, 20 µM glucose-1,6-bisphosphate (Glc-1,6-2P), a range of concentrations of Glc-1-P (60–7,000 µM), 1 mM NAD^+^ and 2 units of G6PDH, the reaction was started by the addition of 56.66 pmol *At*AGM and incubated for 10 min at 30 °C. The results are the mean ± S.D. for three determinations.

In order to identify the effectiveness of this novel method, both traditional coupled assay and HPAEC-PAD method were used to detect the steady-state kinetics and kinetic parameters of *At*AGM. The activity of *Aspergillus fumigates* AGM on Glc-1-P determined by traditional coupled assay from Fang et al. (2013) was also taken as a comparison. The Steady-state kinetics and kinetic parameters of *At*AGM were shown in Fig. 4 and Table 1. The *K_m_* of *At*AGM detected by HPAEC-PAD was 679.18 ± 156.40 µM, which was comparable with the *K_m_* of 707.09 ± 170.36 µM detected by traditional coupled assay. And the *K*_cat_ was also similar between HPAEC-PAD method (40.43 s^−1^) and traditional coupled assay (39.32 s^−1^). These results were comparable with the *K*_cat_ (41.00 s^−1^) obtained from *Af*AGM on the same substrate, which determined by traditional coupled assay (Table 1).

**Table 1:** Kinetic parameters of *At*AGM was analyzed by using Glc-1-P as substrates by two methods. The HPAEC-PAD method was carried out by using 56.66 pmol *At*AGM or boiled enzymes, incubated for 10 min at 30 °C in a substrate buffer (300 µl) consisting of 20 mM PBS, pH 7.0, 10 mM MgSO_4_, and 20 µM glucose-1,6-bisphosphate (Glc-1,6-2P), with varying concentrations of Glc-1-P (60–8,000 µM). The traditional coupled assay was carried out in a 200 µl reaction volume containing 20 mM PBS, pH 7.0, 10 mM MgSO_4_, 20 µM glucose-1,6-bisphosphate (Glc-1,6-2P), a range of concentrations of Glc-1-P (60–7,000 µM), 1 mM NAD^+^ and 2 units of G6PDH, the reaction was started by the addition of 56.66 pmol *At*AGM and incubated for 10 min at 30 °C. The results are the mean ± S.D. for three determinations.

Enzyme	*At*AGM (HPAEC-PAD)	*At*AGM (Coupled assay)	*Af*AGM (Fang et al., 2013)
*K_m_* (µmol L^−1^)	679.18 ± 156.40	707.09 ± 170.36	400.0 ± 30.0
*V*_max_ (nmol s^−1^)	2.29 ± 0.13	2.23 ± 0.15	0.41 ± 0.01(µM∕s)
*K*_cat_ (s^−1^)	40.43	39.32	41.00
*K*_cat_∕*K_m_* (µmol L^−1^ s^−1^)	0.0595	0.0556	0.1025

The HPAEC-PAD method was simpler, more convenient than the traditional coupled assay (Greig et al., 2007; Hofmann, Boles & Zimmermann, 1994). The results obtained by the HPAEC-PAD method and traditional coupled assay were very similar, further confirmed the effectiveness of this novel method. Another significant advantage of HPAEC-PAD method was the substrate consuming and the product increasing could be detected at the same time, which enabled the detection of reverse reaction.

### GlmM assay

Based on the excellent separation and detection abilities of this novel method on all phosphohexoses, we wonder if this method also could be used to determine the activities of GlmM and PGM besides AGM. GlmM is the second enzyme in hexosamine pathway catalyzes the interconvertion of GlcN-6-P and GlcN-1-P in prokaryote (Fig. 1B). The well-studied *Mt*GlmM from *Mycobacterium tuberculosis* was chosen to validate this idea.

*Mt*GlmM activity was calculated as nanomoles of GlcN-6-P or GlcN-1-P consumed at per minute for per milligram protein (Table 2). It is noteworthy that the activity of *Mt*GlmM on GlcN-6-P tested by HPAEC-PAD (7493.40 ± 309.12 nmol∕min ⋅ mg) was much higher than the traditional coupled assay (288.97 ± 35.28 nmol∕min ⋅ mg). One possible reason was that the accuracy of the traditional coupled assay was mainly dependent on the activity of the coupled enzyme GlmU. GlmU is a bifunctional enzyme (Menginlecreulx & Vanheijenoort, 1993; Menginlecreulx & Vanheijenoort, 1994) (Fig. 1B), catalyzed the third and forth steps in the prokaryotic hexosamine pathway. In this coupled system, the activity of GlmM was measured indirectly by detecting the generation of CoA-SH, which was catalyzed by the acetyl transferase activity of GlmU (third step). But the acetyl transferase activity of GlmU has a very strong product inhibition when GlcN-1-P reached 0.003 mM (Singh, Das & Seshadri, 2012), so the GlmU become the rate-limiting enzyme in the traditional coupled assay, which result in the significantly decrease of *Mt*GlmM activity. Furthermore, GlmU was non-commercial, the expression and purification of GlmU was tedious, which makes the process complicated and the results unstable.

**Table 2:** The activity of *Mt*GlmM with GlcN-6-P or GlcN-1-P as substrate was assayed by two methods. The traditional coupled method was assayed in a coupled enzyme system with *Mt*GlmU. The 50 µl reaction mixture contained 50 mM Tris–HCl, pH 8.0, 2.5 mM MgCl_2_, 1 mM GlcN-6-P, 0.6 mM acetyl-CoA, 0.2 mM Glc-1,6-2P, purified *Mt*GlmU (3.5 µg) and purified *Mt*GlmM (0.18 µg). The reaction was incubated at 37 °C for 20 min. The HPAEC-PAD method was carried out by using the purified *Mt*GlmM (0.18 µg), incubated at 37 °C for 20 min in a substrate buffer (50 µl) consisting of 1 mM GlcN-6-P (or GlcN-1-P) as substrate with 50 mM Tris–HCl, pH 8.0, 2.5 mM MgSO_4_, and 0.2 mM Glc-1,6-2P. The results are the mean ± S.D. for three determinations.

Substrates	*Mt*GlmM activity by traditional two-step assay (nmol∕min ⋅ mg)	*Mt*GlmM activity by direct HPLC assay (nmol∕min ⋅ mg)
GlcN-6-P	288.97 ± 35.28	7493.40 ± 309.12
GlcN-1-P	Can’t be measured	6277.90 ± 385.25

In contrast, HPAEC-PAD method could detect the *Mt*GlmM activity directly, which made the detection more sensitive, accurate and convenient. Moreover, HPAEC-PAD method could detect the substrate consuming and the product increasing at the same time, making the detection of reverse reaction possible. The activity of *Mt*GlmM on GlcN-1-P tested by HPAEC-PAD was 6277.90 ± 385.25 nmol∕min ⋅ mg, which comparable with the activity on GlcN-6-P.

## Conclusion

A HPAEC-PAD method was developed to analyze the activity of *α*-D-phosphohexomutases (AGM, PGM and GlmM). This method offers a simple and convenient method to measure the activity of *α*-D-phosphohexomutases on phosphohexoses. It is more sensitive, accurate and reliable than the traditional coupled assay. This method will bring convenience to further research on *α*-D-phosphohexomutases.

10.7717/peerj.1517/supp-1 Supplemental Information 1 Raw data for Fig. 2

10.7717/peerj.1517/supp-2 Supplemental Information 2 Raw data for Fig. 3

10.7717/peerj.1517/supp-3 Supplemental Information 3 Raw data for Fig. 4 and Table 1

10.7717/peerj.1517/supp-4 Supplemental Information 4 Raw data for Table 2

## Supplemental Information

## Abbreviations

AGM: N-acetylglucosamine-phosphate mutase
GlcNAc: N-acetylglucosamine
GlcNAc-1-P: N-acetylglucosamine-1-phosphate
GlcNAc-6-P: N-acetylglucosamine-6-phosphate
GlcN-1-P: Glucosamine-1-phosphate
GlcN-6-P: Glucosamine-6-phosphate
Glc-1-P: Glucose-1-phosphate
Glc-6-P: Glucose-6-phosphate
GlmM: Glucosamine-phosphate mutase
HPAEC-PAD: High-performance anion-exchange chromatography coupled with electrochemical detector
PGM: Phosphoglucomutase

## Additional Information and Declarations

### Data Availability

The following information was supplied regarding data availability: The research in this article did not generate any raw data.
