# Supplementary material for: A simple and rapid method for measuring α-D-phosphohexomutases activity by using anion-exchange chromatography coupled with an electrochemical detector
Source: PeerJ. 2016 Jan 5;4:e1517. doi: 10.7717/peerj.1517 (PMC4715444; doi:10.7717/peerj.1517)

**Table.S1**

**The raw data for Standard curves in Fig.2：**

**GlcNAC-1-P**

| C（mg/ml） | S（nC*min） |
| --- | --- |
| 0.1 | 7.7087 |
| 0.05 | 4.0582 |
| 0.01 | 0.9238 |
| 0.005 | 0.4418 |
| 0.001 | 0.0936 |


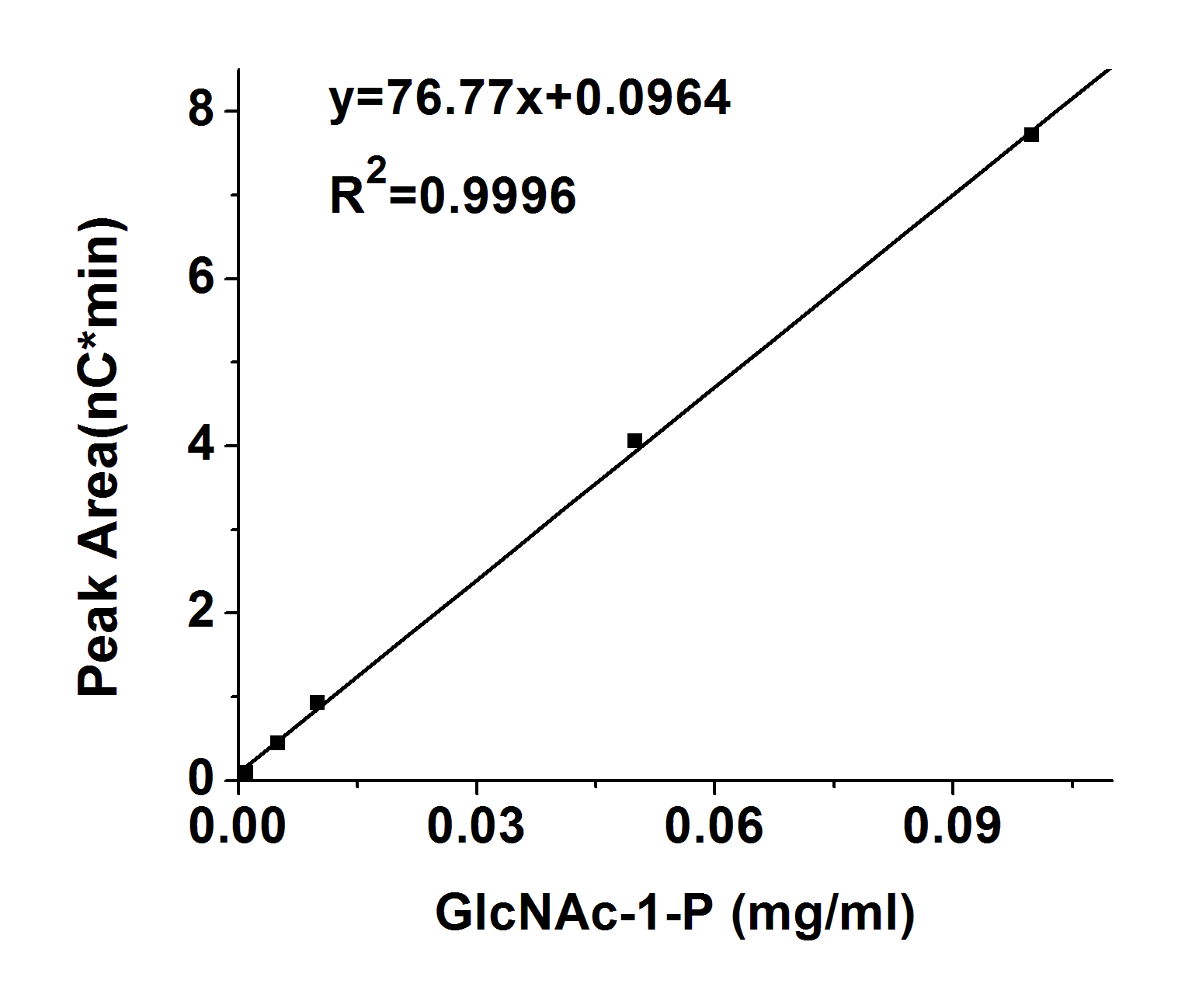


**GlcNAC-6-P**

| C（mg/ml） | S（nc*min） |
| --- | --- |
| 0.1 | 12.6313 |
| 0.05 | 6.4562 |
| 0.01 | 1.7417 |
| 0.005 | 0.8425 |
| 0.001 | 0.1668 |

**
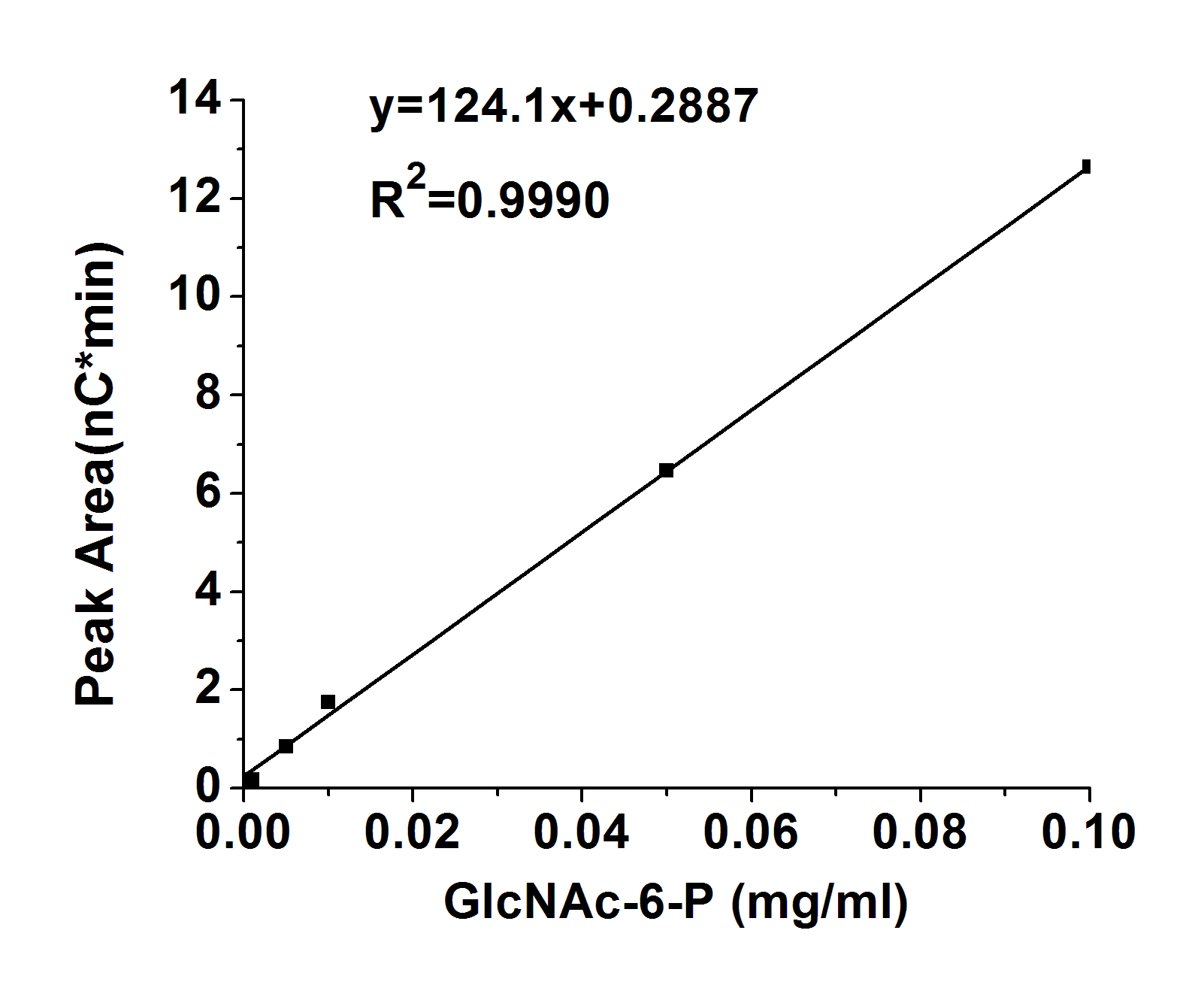
**

**GlcN-1-P**

| C（mg/ml） | S（nc*min） |
| --- | --- |
| 0.1 | 28.7228 |
| 0.01 | 4.2066 |
| 0.001 | 0.4835 |
| 0.05 | 14.3743 |
| 0.005 | 2.0035 |

**
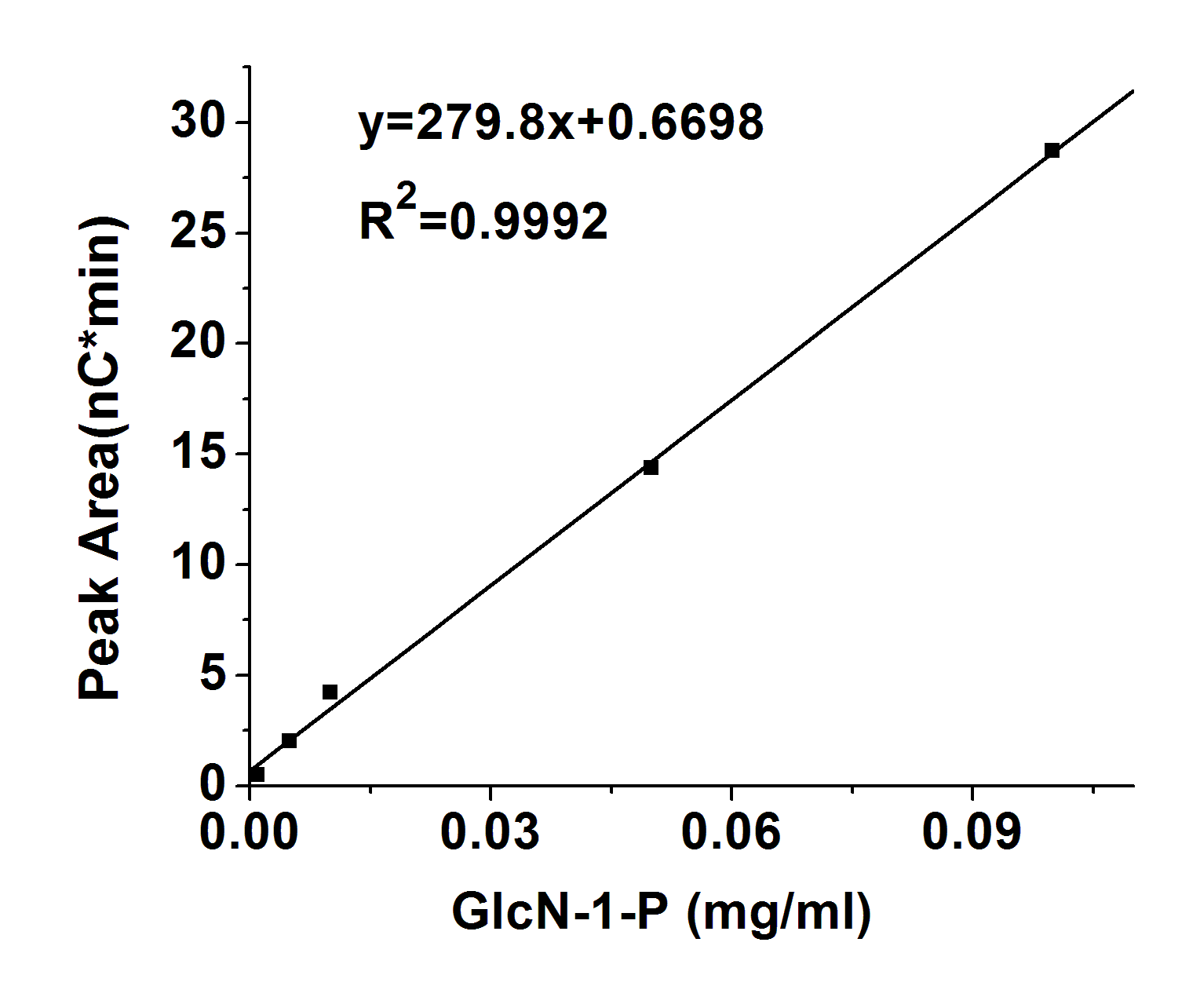
**

**GlcN-6-P**

| C（mg/ml） | S（nc*min） |
| --- | --- |
| 0.1 | 45.1897 |
| 0.01 | 5.2229 |
| 1.00E-03 | 0.5416 |
| 0.05 | 22.3842 |
| 0.005 | 2.4164 |

**
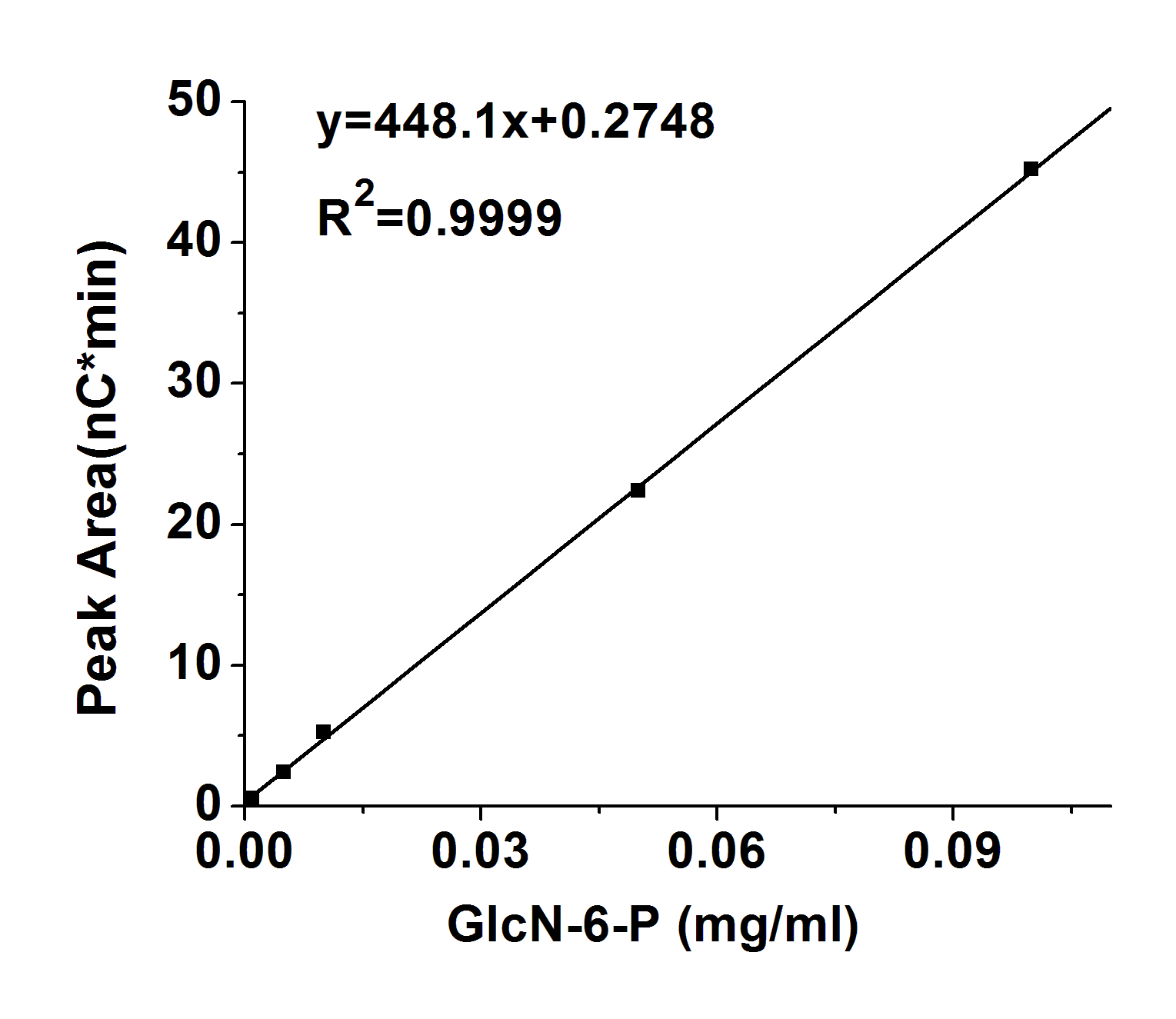
**

**Glc-1-P**

| C（mg/ml） | S（nc*min） |
| --- | --- |
| 0.1 | 11.5466 |
| 0.01 | 1.259 |
| 0.001 | 0.1436 |
| 0.05 | 5.555 |
| 0.005 | 0.5924 |

**
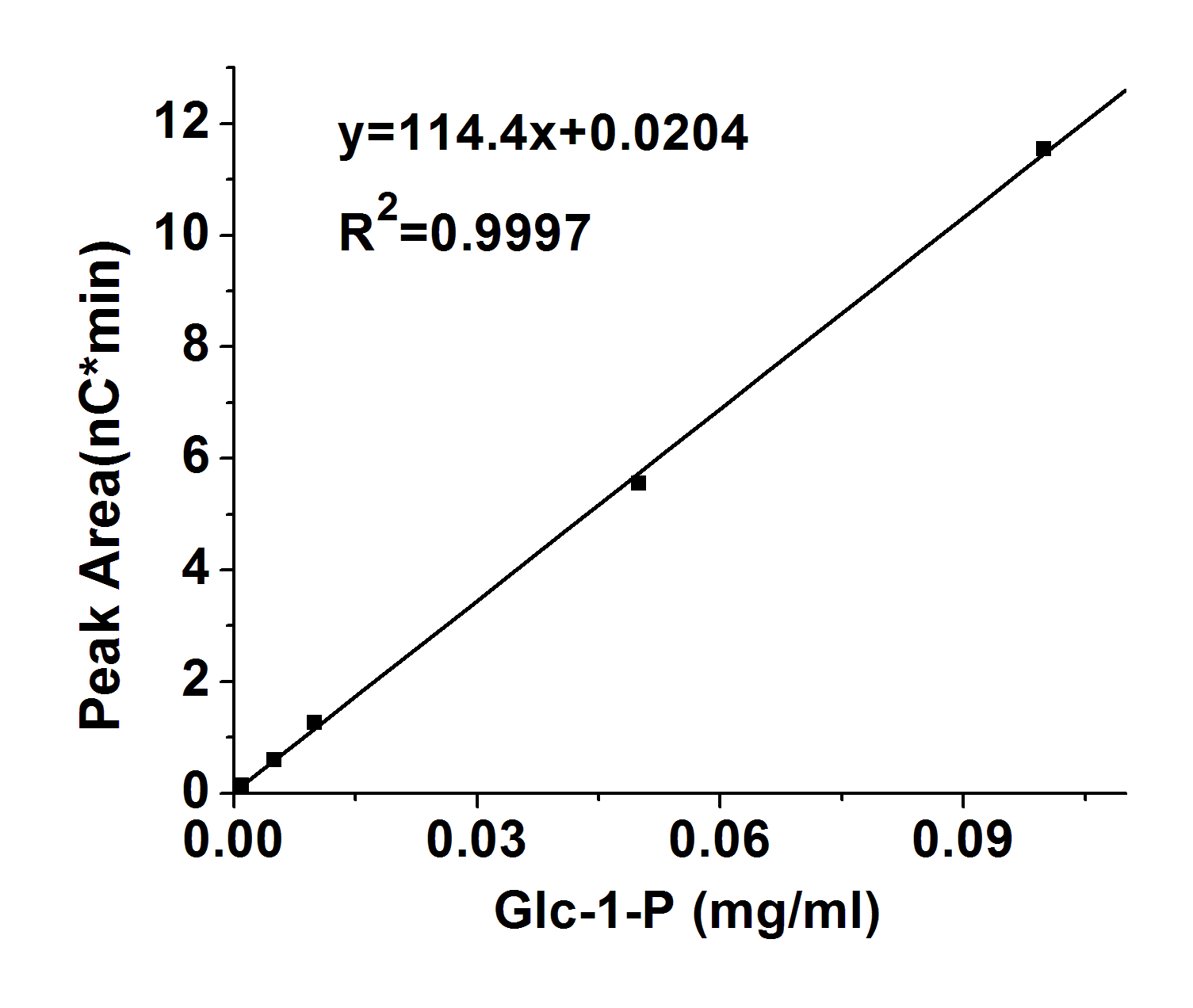
**

**Glc-6-P**

| C（mg/ml） | S（nc*min） |
| --- | --- |
| 0.1 | 18.2927 |
| 0.01 | 1.9931 |
| 0.001 | 0.219 |
| 0.05 | 8.5294 |
| 0.005 | 0.912 |


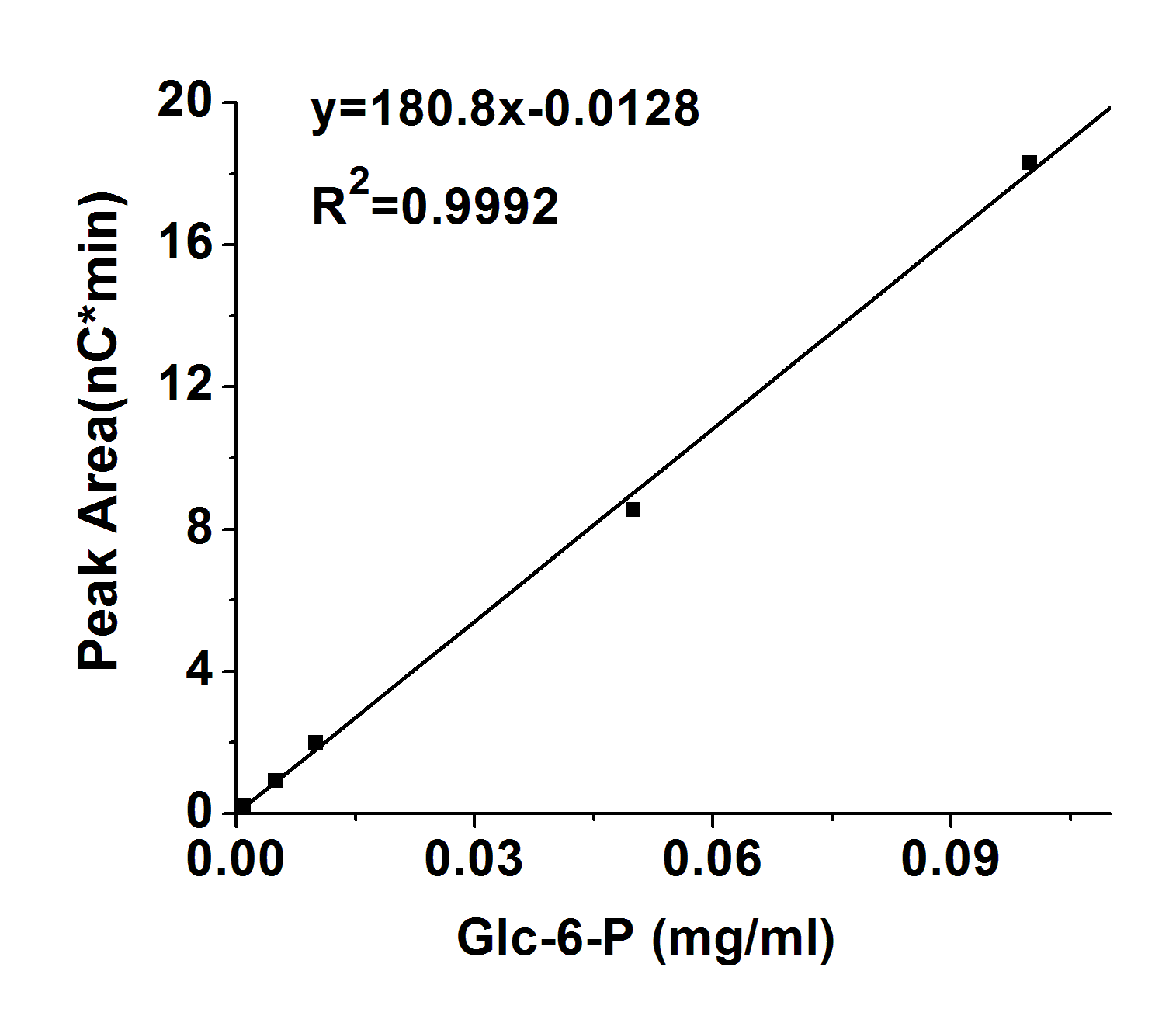

Supplement: Supplemental Information 1 [file peerj-04-1517-s001.docx]
