# Supplementary material for: A simple and rapid method for measuring α-D-phosphohexomutases activity by using anion-exchange chromatography coupled with an electrochemical detector
Source: PeerJ. 2016 Jan 5;4:e1517. doi: 10.7717/peerj.1517 (PMC4715444; doi:10.7717/peerj.1517)
Supplement: Supplemental Information 2 [file peerj-04-1517-s002.docx]

**Fig.S2**

**The raw data for the peak area changes of Chromatograms in Fig.3：**

**Table S2.1 GlcNAc-1-P as substrate,** assayed with 10 μg enzymes using HPAEC after 30 min incubations at 30 ℃.

| Reaction time (min) | GlcNAc-1-P(nC*min) | GlcNAc-6-P(nC*min) |
| --- | --- | --- |
| 0 | 0.4022 | n.a. |
| 30 | 0.0418 | 0.471 |

**Table S2.2 GlcNAc-6-P as substrate,** assayed with 10 μg enzymes using HPAEC after 30 min incubations at 30 ℃.

| Reaction time (min) | GlcNAc-1-P(nC*min) | GlcNAc-6-P(nC*min) |
| --- | --- | --- |
| 0 | n.a. | 0.7297 |
| 30 | 0.0597 | 0.5244 |

**Table S2.3 GlcN-1-P as substrate,** assayed with 10 μg enzymes using HPAEC after 30 min incubations at 30 ℃.

| Reaction time (min) | GlcN-1-P(nC*min) | GlcN-6-P(nC*min) |
| --- | --- | --- |
| 0 | 4.9332 | n.a. |
| 30 | 4.6399 | 0.0715 |

**Table S2.4 GlcN-6-P as substrate**, assayed with 10 μg enzymes using HPAEC after 30 min incubations at 30 ℃.

| Reaction time (min) | GlcN-1-P(nC*min) | GlcN-6-P(nC*min) |
| --- | --- | --- |
| 0 | n.a. | 4.9624 |
| 30 | n.a. | 4.6864 |

**Table S2.5 Glc-1-P as substrate**, assayed with 10 μg enzymes using HPAEC after 30 min incubations at 30 ℃.

| Reaction time (min) | Glc-1-P(nC*min) | Glc-6-P(nC*min) |
| --- | --- | --- |
| 0 | 1.0791 | n.a. |
| 30 | 0.5755 | 0.4702 |

**Table S2.6 Glc-6-P as substrate**, assayed with 30 μg enzymes using HPAEC after 30 min incubations at 30 ℃.

| Reaction time (min) | Glc-1-P(nC*min) | Glc-6-P(nC*min) |
| --- | --- | --- |
| 0 | n.a. | 1.5092 |
| 30 | 0.0283 | 1.2817 |
