# Supplementary material for: A simple and rapid method for measuring α-D-phosphohexomutases activity by using anion-exchange chromatography coupled with an electrochemical detector
Source: PeerJ. 2016 Jan 5;4:e1517. doi: 10.7717/peerj.1517 (PMC4715444; doi:10.7717/peerj.1517)
Supplement: Supplemental Information 3 [file peerj-04-1517-s003.docx]

**The raw data for Fig.4 and table.1**

The steady-state kinetics of *At*AGM was detected by two methods (using Glc-1-P as substrates). When using the low concentration Glc-1-P as substrates (60μM), the Glc-1-P consuming (HPAEC-PAD method, 3.66 μg *At*AGM) and NADH increasing (Traditional coupled assay, 3.66 μg *At*AGM) were shown in Supplement Fig. 1(A, B). We chose 10min as reaction time because the reaction was still in initial rate stage.

**Supplement Fig. 1**


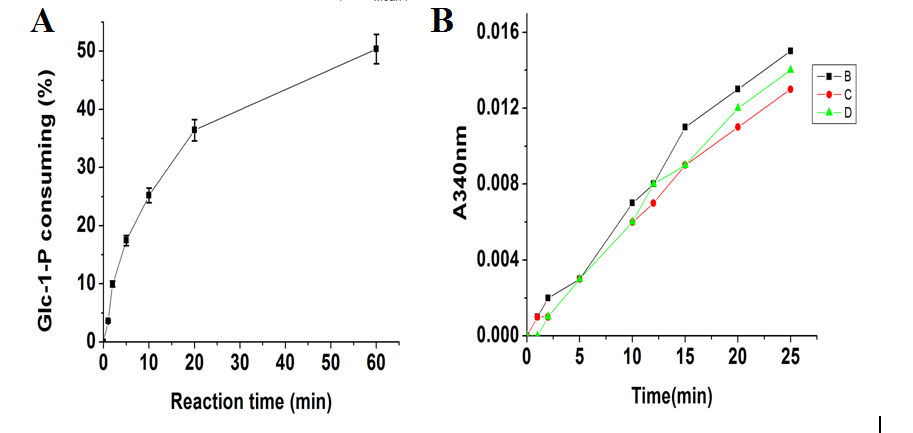


About three μg *At*AGM protein or boiled enzymes (3.66 μg for HPAEC-PAD method, 3.15 μg for traditional coupled assay), were incubated for 10 min at 30 ℃ in a substrate buffer as described in the article, with varying concentrations of Glc-1-P (60-8000 μM). The results are the mean ± S.D. for three determinations.

**Supplement table.1**

HPAEC-PAD method

| **C(uM)** | **V(nmol/s)-1** | **V-2** | **V-3** |
| --- | --- | --- | --- |
| 8023.67642 | 2.700334128 | 1.319841 | 2.217628 |
| 6017.75732 | 2.52298617 | 1.301063 | 2.57834 |
| 4011.83821 | 2.345148511 | 1.431038 | 2.538697 |
| 2005.91911 | 1.823549285 | 0.932758 | 1.667311 |
| 802.36764 | 0.979258511 | 1.032893 | 1.534254 |
| 401.18382 | 0.813657574 | 0.869624 | 1.039853 |
| 80.23676 | 0.401136 | 0.398396 | 0.400314 |
| 60.17757 | 0.300852 | 0.295372 | 0.298934 |

Using the data shown above, we could match with michaelis-menten equation by using Oringin 7.5. The relative Steady-state kinetics data was shown in Table.1.

**Supplement table.2**

| **C(uM)** | **V(nmol/s)-1** | **V-2** | **V-3** |
| --- | --- | --- | --- |
| 6889.181 | 1.557054 | 1.938726 | 2.186885 |
| 4814.206 | 1.850766 | 1.817522 | 2.056786 |
| 1805.327 | 1.993174 | 2.020397 | 1.626686 |
| 481.4206 | 0.932975 | 0.869363 | 0.890567 |
| 300.8879 | 0.594915 | 0.571303 | 0.551303 |
| 180.5327 | 0.33806 | 0.360468 | 0.319264 |
| 60.17757 | 0.148428 | 0.127224 | 0.127224 |

Using the data shown above, we could match with michaelis-menten equation by using Oringin 7.5. The relative Steady-state kinetics data was shown in Table.1.
