# Supplementary material for: A simple and rapid method for measuring α-D-phosphohexomutases activity by using anion-exchange chromatography coupled with an electrochemical detector
Source: PeerJ. 2016 Jan 5;4:e1517. doi: 10.7717/peerj.1517 (PMC4715444; doi:10.7717/peerj.1517)
Supplement: Supplemental Information 4 [file peerj-04-1517-s004.docx]

**The raw data for table.2**

The activity of *Mt*GlmM was detected by HPAEC-PAD method and traditional coupled assay.

**1---HPAEC-PAD method**

**Using GlcN-6-P or GlcN-1-P as substrate,** 0.18 μg *Mt*GlmM were inoculated in the reaction buffer described in the article at 37℃ for 20 min (in initial rate stage). The raw data are shown in Supplement table.1 and Supplement table.2.

**Supplement table.1**

**Using GlcN-1-P as substrate,** the activity of *Mt*GlmM was detected by HPAEC-PAD method. And “T” means retention time，“consumed” means the substrate consumed compared with 0min, and the last list was the activity data of *Mt*GlmM detected using HPAEC-PAD.

|  | **T(min)** | **S(nC*min)** | **C(mg/mL)** | **Consumed（mg/mL）** | **Consumed（%）** | **nmol/mg.min** |
| --- | --- | --- | --- | --- | --- | --- |
| 1-0 | 11.734 | 0.4026 | 0.02694 |  |  |  |
| 1-0 | 11.734 | 0.39 | 0.025966 |  |  |  |
| 1-1 | 11.767 | 0.2502 | 0.015162 | 0.011288 | 42.67566 | 6654.492128 |
| 1-2 | 11.767 | 0.2581 | 0.015773 | 0.010677 | 40.3675 | 6294.5756 |
| 1-3 | 11.8 | 0.2671 | 0.016468 | 0.009982 | 37.73794 | 5884.544112 |

**Supplement table.2**

**Using GlcN-6-P as substrate,** the activity of *Mt*GlmM was detected by HPAEC-PAD method. And “T” means retention time，“consumed” means the substrate consumed compared with 0min, and the last list was the activity data of *Mt*GlmM detected using HPAEC-PAD.

|  | **T(min)** | **S(nC*min)** | **C(mg/mL)** | **Consumed（mg/mL）** | **Consumed（%）** | **nmol/mg.min** |
| --- | --- | --- | --- | --- | --- | --- |
| 6-0 | 14.067 | 1.0884 | 0.03346 |  |  |  |
| 6-1 | 14.067 | 0.7282 | 0.021192 | 0.012268 | 36.66438 | 7232.347 |
| 6-2 | 14.084 | 0.7192 | 0.020886 | 0.012574 | 37.58052 | 7413.063 |
| 6-3 | 14.1 | 0.6982 | 0.02017 | 0.01329 | 39.71817 | 7834.732 |

**2---Traditional coupled assay**

**Using GlcN-6-P as substrate,** 0.18 μg *Mt*GlmM were inoculated in the reaction buffer described in the article at 37℃ for 20 min (in initial rate stage). The reaction buffer details and raw data were shown in Supplement table.3 and Supplement table.4.

**Supplement table.3**

**The reaction buffer details** for the detection of the activity of *Mt*GlmM by traditional coupled assay. The reaction was incubated at 37℃ for 20 min and terminated by adding 50 μl of stop solution containing 50 mM Tris-HCl, pH 7.5, 6 M guanidine hydrochloride.

|  | **blank** | **standard** | ***Mt*GlmM-1** | ***Mt*GlmM-2** | ***Mt*GlmM-3** |
| --- | --- | --- | --- | --- | --- |
| 1M Tris-HCl (PH 8.0) (ul) | 2.5 | 2.5 | 2.5 | 2.5 | 2.5 |
| 50 mM MgSO_4_(ul) | 2.5 | 2.5 | 2.5 | 2.5 | 2.5 |
| 10 mM GlcN-6-P (ul) | - | - | 5.7 | 5.7 | 5.7 |
| 10 mM AC-CoA (ul) | - | - | 3 | 3 | 3 |
| 1 mM CoA (ul) | - | 2 | - | - | - |
| 10 mM Glc-1,6-diP(ul) | - | - | 1 | 1 | 1 |
| 0.06 mg/ml GlmM (ul) | - | - | 3 | 3 | 3 |
| 0.69 mg/ml GlmU (ul) | - | - | 5 | 5 | 5 |
| ddH_2_O (ul) | 45 | 43 | 27.3 | 27.3 | 27.3 |

**Supplement table.4**

**Using GlcN-6-P as substrate,** the activity of *Mt*GlmM was detected by traditional coupled assay.

|  | **blank** | **standard** | ***Mt*GlmM-1** | ***Mt*GlmM-2** | ***Mt*GlmM-3** |
| --- | --- | --- | --- | --- | --- |
| A_405_ | 0.0971 | 0.2898 | 0.1885 | 0.1922 | 0.2113 |
| -blank |  | 0.1927 | 0.0914 | 0.0951 | 0.1142 |
| CoA produced (nmol) |  | 2 | 0.948624805 | 0.987026466 | 1.185262065 |
| nmol/min·mg |  |  | 263.5068904 | 274.1740183 | 329.2394626 |
